# Prediction of the VeriStrat test in first-line therapy of pemetrexed-based regimens for advanced lung adenocarcinoma patients

**DOI:** 10.1186/s12935-020-01662-5

**Published:** 2020-12-09

**Authors:** Bo Jia, Zhi Dong, Di Wu, Jun Zhao, Meina Wu, Tongtong An, Yuyan Wang, Minglei Zhuo, Jianjie Li, Yang Wang, Jie Zhang, Xinghui Zhao, Sheng Li, Junfeng Li, Menglei Ma, Chen Chen, Xue Yang, Jia Zhong, Hanxiao Chen, Jingjing Wang, Yujia Chi, Xiaoyu Zhai, Song Cui, Rong Zhang, Qingwei Ma, Jian Fang, Ziping Wang

**Affiliations:** 1 Key laboratory of Carcinogenesis and Translational Research (Ministry of Education/ Beijing), Department of Thoracic Medical Oncology, Peking University Cancer Hospital & Institute, 52 Fucheng Road, Haidian District, Beijing, 100142 China; 2 Key laboratory of Carcinogenesis and Translational Research (Ministry of Education/ Beijing), Department of GI Oncology, Peking University Cancer Hospital & Institute, Beijing, China; 3 Key Laboratory of Carcinogenesis and Translational Research (Ministry of Education/Beijing), Department of Thoracic Oncology II, Peking University Cancer Hospital & Institute, 52 Fucheng Road, Haidian District, 100142 Beijing, China; 4 Center for Clinical Laboratory Medicine, Chinese PLA General Hospital, The First Medical Center), Beijing, China; 5 Bioyong Technologies Inc, Beijing, China

**Keywords:** Lung adenocarcinoma, Pemetrexed, Prognosis, Treatment, VeriStrat

## Abstract

**Background:**

Although advanced non-squamous non-small cell lung cancer (NSCLC) patients have significantly better survival outcomes after pemetrexed based treatment, a subset of patients still show intrinsic resistance and progress rapidly. Therefore we aimed to use a blood-based protein signature (VeriStrat, VS) to analyze whether VS could identify the subset of patients who had poor efficacy on pemetrexed therapy.

**Methods:**

This study retrospectively analysed 72 advanced lung adenocarcinoma patients who received first-line pemetrexed/platinum or combined with bevacizumab treatment.

**Results:**

Plasma samples from these patients were analysed using VS and classified into the Good (VS-G) or Poor (VS-P) group. The relationship between efficacy and VS status was further investigated. Of the 72 patients included in this study, 35 (48.6%) were treated with pemetrexed plus platinum and 37 (51.4%) were treated with pemetrexed/platinum combined with bevacizumab. Among all patients, 60 (83.3%) and 12 (16.7%) patients were classified as VS-G and VS-P, respectively. VS-G patients had significantly better median progression-free survival (PFS) (Unreached vs. 4.2 months; P < 0.001) than VS-P patients. In addition, the partial response (PR) rate was higher in the VS-G group than that in the VS-P group (46.7% vs. 25.0%, P = 0.212). Subgroup analysis showed that PFS was also significantly longer in the VS-G group than that in the VS-P group regardless of whether patients received chemotherapy alone or chemotherapy plus bevacizumab.

**Conclusions:**

Our study indicated that VS might be considered as a novel and valid method to predict the efficacy of pemetrexed-based therapy and identify a subset of advanced lung adenocarcinoma patients who had intrinsic resistance to pemetrexed based regimens. However, larger sample studies are still needed to further confirm this result.

## Introduction

Lung cancer is the most common cancer worldwide, and most patients are diagnosed at an advanced stage [1, 2]. Over the past decades, although the treatment for non-small cell lung cancer (NSCLC) patients has notably improved, significant progress has been made in immunotherapy and targeted therapy, but chemotherapy remains the cornerstone for advanced NSCLC patients.

Pemetrexed-based therapy has been the standard first-line chemotherapy regimen for advanced non-squamous NSCLC patients without epidermal growth factor receptor (EGFR)-sensitizing mutations, anaplastic lymphoma kinase (ALK) or c-ros oncogene 1 receptor kinase (ROS1) rearrangement. First-line pemetrexed-based chemotherapy showed significantly better outcomes than other chemotherapy regimens for these patients, especially gemcitabine/platinum [3–5]. Bevacizumab is a recombinant humanized monoclonal antibody that can bind vascular endothelial growth factor-A (VEGF-A), inhibit its binding to VEGF receptor-2, and inhibit the biological effects of VEGF. As an anti-angiogenesis targeted drug, bevacizumab has been approved for the first-line treatment of advanced, metastatic or recurrent non-squamous NSCLC patients [6]. However some patients still showed primary resistance to these treatment regimens and progressed rapidly. It is unclear why these patients responded heterogeneously and it is necessary to find a method to predict the efficacy and identify a subset of patients who might be resistant to pemetrexed-based chemotherapy alone or combined with bevacizumab. Therefore these patients would have a chance to receive other treatment methods, such as immunotherapy, targeted therapy, and other chemotherapy regimens.

VeriStrat (VS), a blood-based test can divide patients into either VS Good (VS-G) or VS Poor (VS-P) groups, which might potentially help oncologists make decisions in their clinical practice. Previous studies have shown that VS is a valid method to predict the efficacy of chemotherapy or targeted therapy for advanced NSCLC patients [7–11]. However, to date, there has not been a study using the VS method for the prediction of first-line pemetrexed based chemotherapy plus for Chinese advanced lung adenocarcinoma patients without EGFR sensitizing mutations or ALK or ROS1 rearrangement. Therefore we conducted a study to explore whether VS could be used in the first-line setting to identify advanced adenocarcinoma patients who had better outcomes or resistance to pemetrexed chemotherapy.

## Materials and methods

###  Patents

Patients were enrolled in this study if they had stage IIIB or IV lung adenocarcinoma, had no previous systemic anticancer therapy and had measurable lesions. All patients received chemotherapy or bevacizumab. Chemotherapy regimens included pemetrexed (500 mg/m^2^ q21d) in combination with cisplatin (75 mg/m^2^ q21d) or carboplatin (AUC = 5 q21d). The dose of bevacizumab was 7.5 mg/kg, q21d. The primary endpoint was progression-free survival (PFS). Other endpoints included partial response and objective response rate (ORR). PFS was calculated from the date of initiation of chemotherapy to the date of progression or death from any cause. The tumour response was assessed by RECIST version 1.1.

### Samples and VeriStrat test

Serum samples were collected and stored frozen. Samples were anonymized and shipped to Bioyong (Beijing,China). VeriStrat analysis was conducted on 72 serum samples by Bioyong (Beijing, China) who was blinded to the clinical and treatment data. Matrix-assisted laser desorption ionization time of flight mass spectrometry (MALDI-TOF-MS) is a new type of soft ionization biomass spectrometry developed in recent years and is the most commonly used frontier technology in proteomics research. MALDI-TOF mass spectrometry can be used to detect protein polypeptide components in patients’ serum, and the molecular weight and content could be portrayed as protein polypeptide fingerprints. VeriStrat (VS) could eventually discover and capture new specific protein features by comparing the differences in atlases between different case groups and by bioinformatics analysis. VeriStrat testing was performed as described [7, 12]. This test used the Clin-TOFII mass spectrometer(based on MALDI mass spectrometry) (Bioyong, Beijing, China). Samples were thawed on ice and diluted 1:10 in HPLC-grade water and then combined with an equal volume of matrix solution.(25 mg/mL sinapinic acid prepared in 50% acetonitrile/0.1% trifluoroacetic acid). A total of 2 µL of sample-matrix mixture was spotted on polished stainless steel MALDI plates three times. Data was acquired on the Clin-TOFII mass spectrometer (Bioyong,  Beijing, China) in linear mode. ASCII files were exported from the spectra, and the VeriStrat algorithm was run on the ASCII files. A VeriStrat label of Good or Poor was designed for each sample when all replicates from a sample produced the same classification. An indeterminate classification status was assigned to samples with discordant findings in the replicates. Only patients with classifications of VeriStrat good (VS-G) or VeriStrat Poor(VS-P) were included in this study cohort.

### Statistical analysis

Baseline characteristics between the VS-G and VS-P groups were compared using a T test for age and the X^2^ test for all other variables. Tumour response was compared using the X^2^ test in two groups. PFS was compared using univariate analysis. A Cox model was used to adjust differences in PFS between groups for other confounding variables (gender, stage, smoking status, and treatment).

## Results

### Patient characteristics

Table 1 shows the patients’ baseline clinical characteristics in this study. Of the 72 patients, 60 (83.3%) were classified as VS-G and 12 (16.7%) as VS-P. The median age was 58 years old in both groups (P = 0.682). Patients’ baseline characteristics were well balanced between the VS-G and VS-P groups (P = 1.000 for gender, P = 1.000 for performance status, P = 1.000 for stage, P = 0.595 for smoking status). Twenty-nine patients (48.3%) in the VS-G group and 6 patients (50.0%) in the VS-P group received chemotherapy alone, with 31 (51.7%) in the VS-G group and 6 patients (50%) in the VS-P group received chemotherapy plus bevacizumab (P = 0.916). The patients’ mass spectrometry signature are provided in original data.

**Table 1:** Patients’ characteristics according to VS classification

	All patients (%)	VS-G (%)	VS-P (%)	P^a^
Age (years)				0.682
Median (range)	58 (34–81)	58 (34–81)	58 (46–74)	
Gender				1.000
Female	24 (33.3)	20 (33.3)	4 (33.3)	
Male	48 (66.7)	40 (66.7)	8 (66.7)	
ECOG PS				1.000
0	42 (58.3)	35 (58.3)	7 (58.3)	
1	30 (41.7)	25 (41.7)	5 (41.7)	
Stage				1.000
IIIB	14 (19.4)	12 (20.0)	2 (16.7)	
IV	58 (80.6)	48 (80.0)	10 (83.3)	
Smoking				0.595
Yes	41 (56.9)	35 (58.3)	6 (50.0)	
No	31 (43.1)	25 (41.7)	6 (50.0)	
Treatment				0.916
Chemotherapy	35 (48.6)	29 (48.3)	6 (50.0)	
Chemotherapy + Bev	37 (51.4)	31 (51.7)	6 (50.0)	

*ECOG* Eastern Cooperative Oncology Group, *PS* performance status, *Bev* bevacizumab. ^a^Two groups were compared using T test for age or using X^2^ test for all other characteristics

### Tumour response

Table 2 illustrates the tumour response in the two groups. The partial response (PR) rate was higher in the VS-G group than in the VS-P group (46.7% vs. 25.0%, P = 0.212). There were no significant differences in the stable disease (SD) rate (P = 0.343) or progression disease (PD) rate (P = 0.526) between the VS-G and VS-P groups. For patients who received chemotherapy alone, the PR rates were 31.0% and 0.0% in the VS-G and VG-P groups respectively. There were no significant differences in the SD rate (P = 0.377) or PD rate (P = 0.442) between the two groups. For patients who underwent chemotherapy and bevacizumab treatment, the PR rate was also higher in the VS-G than that in the VG-P group (61.3% for VS-G vs. 50.0% for VS-P). The SD rates were 35.5% and 50.0% (P = 0.653) in the VS-G and VS-P groups, respectively, with PD rates of 3.2% and 0.0% (P = 1.000), respectively. The difference did not reach statistical significance, likely because of the limited number of patients in this study (Table 2).

**Table 2:** Tumor response according to VS Classification

	VS-G (%)	VS-P (%)	P^a^			
PR	28 (46.7)	3 (25.0)	0.212			
SD	28 (46.7)	8 (66.7)	0.343			
PD	3 (5.0)	1 (8.3)	0.526			
PR+SD	56 (93.3)	11 (91.7)	1.000			

	Chemotherapy VS-G(%)	VS-P(%)	P^a^	Chemotherapy+Bev VS-G(%)	VS-P(%)	P^a^
PR	9 (31.0)	0 (0.0)	0.304	19 (61.3)	3 (50.0)	0.670
SD	17 (58.6)	5 (83.3)	0.377	11 (35.5)	3 (50.0)	0.653
PD	2 (6.9)	1 (16.7)	0.442	1 (3.2)	0 (0.0)	1.000
PR+SD	26 (89.7)	5 (83.3)	0.546	30 (96.8)	6 (100.0)	1.000

*PR* partial response, *SD* stable disease, *PD* progression disease, *Bev* bevacizumab

^a^Tumor response was compared using X^2^ test in two groups

### Survival

**Fig. 1:**
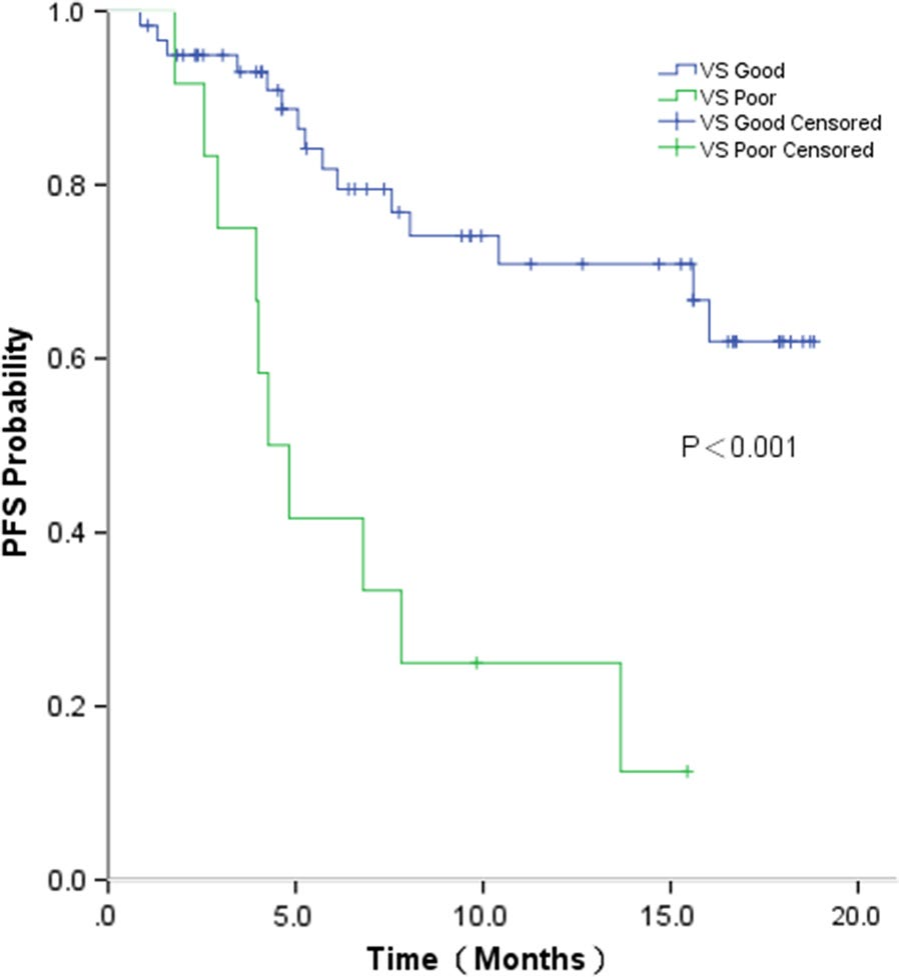
Progression-free survival (PFS) by VS classification for all patients

**Fig. 2:**
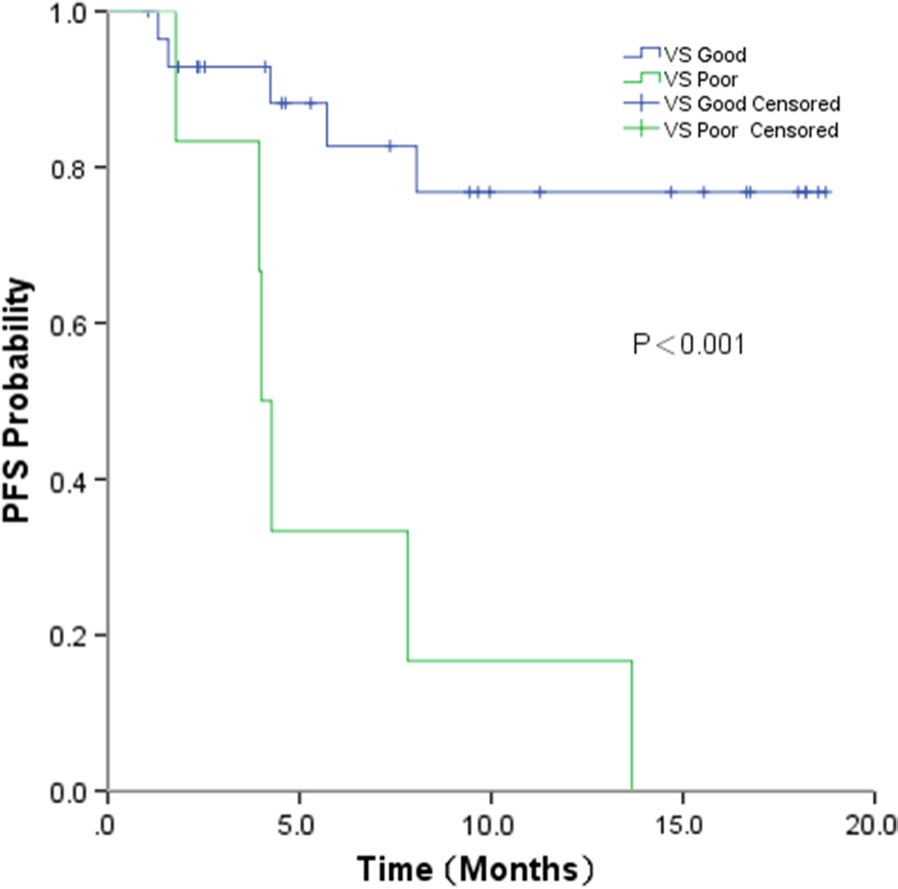
Progression-free survival (PFS) by VS classification for patients received chemotherapy alone

**Fig. 3:**
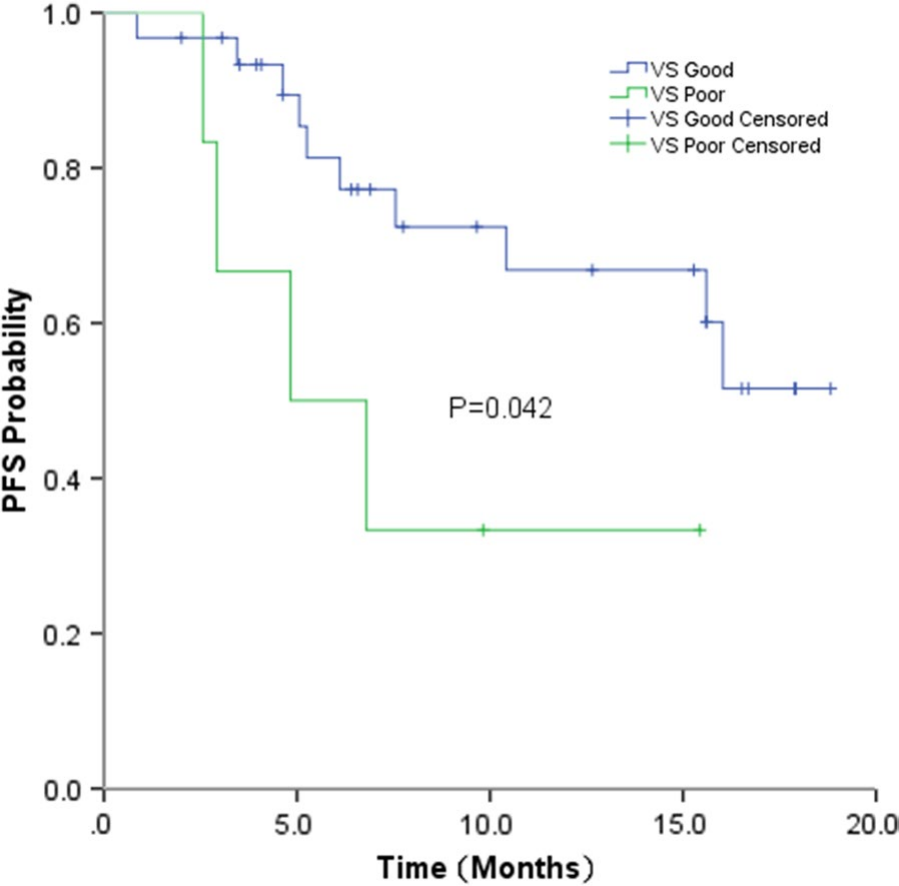
Progression-free survival (PFS) by VS classification for patients received chemotherapy and bevacizumab

Of 72 patients in this study, 25 patients experienced disease progression. The median follow-up time for all patients was 7.4 months (0.9–18.8 months). A significantly improved median PFS was observed for patients in the VS-G group compared with that in the VS-P group (Unreached vs. 4.2 months; P < 0.001) (Fig. 1). Median OS was not reached in either group. For the 35 patients who received chemotherapy, the median PFS was significantly higher for patients in the VS-G group than that in patients in the VS-P group (Unreached vs. 4.0 months; P < 0.001) (Fig. 2). For 37 patients treated with chemotherapy and bevacizumab, the median PFS was also significantly longer in the VS-G group than that in the VS-P group (Unreached vs. 4.8 months, p = 0.042) (Fig. 3). Patients who did not relapse or progress at the last follow-up were referred to as censored data. The interaction between PFS and VS classification was tested using the Cox model. After adjusting for gender, stage, smoking status and treatment by multivariate analysis, the interaction between PFS and VS classification was also statistically significant (P = 0.011) (Table 3).

**Table 3:** Association of treatment with progression-free survival (PFS)

	Univariate Analysis	Multivariate Analysis	
	P ^a^	HR (95% C.I.)	P^b^
Treatment			
Chemotherapy vs. chemotherapy + bevacizumab	0.890	0.734 (0.325–1.656)	0.457
Gender			
Male vs. female	0.344	0.998 (0.262–3.803)	0.998
Smoking status			
Smoking vs. non-smoking	0.379	0.646 (0.201–2.077)	0.463
Stage			
IV vs. IIIB	0.059	0.265 (0.061–1.150)	0.076
VeriStrat			
Good vs. poor	< 0.001	0.214 (0.088–0.522)	0.011

*HR* hazard ratio, *CI*, confidence interval

^a^P-value was estimated by univariate analysis

^b^HR, 95% C.I. and P-value were estimated in Cox proportional hazards model

## Discussion

This was the first study indicating that a blood-based protein signature (VS) might be a novel and valid method to predict the efficacy of pemetrexed/platinum or bevacizumab in first-line treatment for Chinese advanced lung adenocarcinoma patients.

The concept of combination therapy refers to using the best combination of existing treatment methods according to the specific conditions of patients, such as body condition, pathological type, invasion range (pathological stage) and development trend, to greatly improve the cure rate, prolong the survival period and improve the quality of life of patients. Surgery, radiotherapy, and systemic therapy are the 3 modalities of combination therapy for cancer patients. Systemic therapy, including chemotherapy, targeted therapy and immunotherapy, is suitable for the treatment of advanced NSCLC patients. Recently, although significant progress has been made in immunotherapy and targeted therapy, chemotherapy remains the cornerstone for advanced NSCLC patients [13–20]. Pemetrexed is a third-generation cytotoxic agent. It can inhibit cell replication and tumour growth by disrupting folate-dependent normal cellular metabolism. Several clinical trials demonstrated that first-line pemetrexed-based therapy was associated with significantly better survival outcomes than other chemotherapy regimens for advanced lung adenocarcinoma patients. In addition, the combination of bevacizumab could further improve survival for these patients. Therefore, pemetrexed/platinum combined with bevacizumab has been the standard first-line chemotherapy regimen for advanced non-squamous NSCLC patients. However, some patients still showed poor response to pemetrexed-based regimens [21, 22]. Currently, due to the lack of clinical evidence, no specific biomarkers have been applied in clinical practice. Therefore, promising biomarkers are urgently needed to predict the efficacy of cytotoxic agents.

Protein polypeptide distributions varied significantly in advanced lung adenocarcinoma patients’ serum, and these protein polypeptides could be detected in different molecular weight ranges by using MALDI-TOF-MS. Eight characteristic peaks in the serum were found to be related to the efficacy of chemotherapy. The height and area of characteristic peaks in the mass spectra of patients with good efficacy were lower, while those with poor efficacy were significantly higher. According to the location, height and area of characteristic peaks, data models were established to predict the efficacy. After comparing the sensitivity and specificity of different models, the VeriStrat test was the model with the best sensitivity and specificity. As a blood-based test, VS could effectively divide patients into either good efficacy (VS-G) or poor efficacy (VS-P) groups. In 2007, David Carbone et al. initially used the VeriStrat method to predict the first-line efficacy of EGFR tyrosine kinase inhibitors (TKIs) for advanced NSCLC patients who did not receive EGFR mutation tests before treatment. In this study, the median survival was 306 days in the VS-G group of 69 patients, far more than the 107 days in the VS-P group of 27 patients [4]. A series of follow-up studies demonstrated that VS is a predictor of therapeutic benefit from EGFR TKI therapy [23]. In the PROSE study, the VS method was utilized to predict second-line single-drug chemotherapy for advanced lung cancer (pemetrexet/docetaxel). The results show that among the 129 patients receiving single-drug chemotherapy, the OS and PFS were significantly lower in the VS-P group than those in the VS-G group [24]. Another study examined the performance of VeriStrat in three independent clinical trials from 481 patients treated with platinum-based chemotherapy as a first-line treatment. Patients classified as VS-G had significantly longer PFS and OS than VS-P patients. These results demonstrated that VS is a strong predictive test in NSCLC patients treated with platinum-based regimens in the first line [10]. However, to date, there has been no study using this VS method for the prediction of first-line pemetrexed plus platinum-based chemotherapy or combined with bevacizumab for Chinese advanced lung adenocarcinoma patients.

To address these issues, we conducted a retrospective analysis of plasma samples from lung adenocarcinoma patients with stage IIIB or IV disease who received first-line pemetrexed-based chemotherapy. Our study showed that median PFS was unreached in the VS good group and was significantly superior to that in VS poor group, the PFS of which was 4.2 months. For the 35 patients who received chemotherapy, an improved PFS was still observed for patients in the VS-G vs. VS-P group (median PFS: Unreached vs. 4.0 months). A recent study included 76 non-squamous patients treated with a combination of carboplatin or cisplatin with pemetrexed. Patients classified as VS-G had longer PFS and OS than VS-P: 6.5 vs. 1.6 months and 10.8 vs. 3.4 months, respectively [11]. The PFS in our study was longer than that found in previously reported data, likely because pemetrexed maintenance therapy was administered in our study. The PARAMOUNT study demonstrated that continuation maintenance therapy with pemetrexed was an effective and well-tolerated treatment option for patients with advanced non-squamous NSCLC with good performance status who did not progress after induction therapy with pemetrexed plus cisplatin [25]. In the PARAMOUNT study, the median PFS was 4.1 months for pemetrexed and 2.8 months for placebo [26]. The result in our study was consistent with that in the PARAMOUNT study.

In the POINTBREAK study, PFS was significantly improved with pemetrexed/carboplatin plus bevacizumab and pemetrexed/bevacizumab maintenance therapy (median PFS, 6.0 vs. 5.6 months; P = 0.012) [6]. In the AVAPEAL study, bevacizumab plus pemetrexed maintenance was also associated with a significant PFS benefit compared with bevacizumab alone (median, 3.7 vs. 7.4 months; P < 0.001) [27]. Updated survival analysis of the AVAPERL study showed that maintenance with bevacizumab-pemetrexed was associated with a nonsignificant increase in OS over bevacizumab alone [28]. A recent study (WJOG 5610L) reported in 2019 that the American Society of Clinical Oncology annual meeting demonstrated that bevacizumab and pemetrexed maintenance therapy could not prolong OS compared with bevacizumab maintenance therapy alone (median, 23.3 vs. 19.6 months; P = 0.069). In our study, for 37 patients treated with chemotherapy and bevacizumab, PFS was also significantly longer in the VS-G group than in the VS-P group (median PFS: Unreached vs. 4.8 months). Our study indicated that VS is also predictive for chemotherapy and bevacizumab combined therapy. Although this study clearly identified patients who might have worse outcomes on pemetrexed-based therapy, these data were not compelling enough to deny pemetrexed therapy to VS-P patients. However, perhaps in these VS-P patients, alternative treatment approaches could be considered.

Several limitations of our study are worthy of note. First, 72 patients were eligible for inclusion and analysis, and only a small subset of participants (12/72, 16.7%) tested as VS-P. This limited the power of the analysis we performed. Furthermore, OS data were not available. Because OS was typically not calculated until more than 50% of patients experienced events, median OS was not reached in either group. Third, the sample number was small, so it was difficult to avoid selective bias. However, we took a series of measures to reduce bias. Patients were consecutively included in this study to avoid selective bias. Patients’ baseline characteristics were compared using the T test for age and the X^2^ test for all other characteristics. We found that patient characteristics were well balanced between the VS-G and VS-P groups. In addition, we used the Cox proportional hazards model to conduct multivariate analysis. After adjusting for gender, stage, smoking status and treatment by multivariate analysis, the interaction between PFS and VS classification was also statistically significant.

## Conclusions

Our study indicated that VS might be considered a novel and valid method to predict the efficacy of pemetrexed-based therapy and identify a subset of advanced lung adenocarcinoma patients who had intrinsic resistance to pemetrexed based regimens. However, larger sample studies are still needed to further confirm our results.

## Data Availability

The raw data supporting the conclusions of this manuscript will be made available by the authors, without undue reservation, to any qualified researcher.
